# The Expression of Serglycin Is Required for Active Transforming Growth Factor β Receptor I Tumorigenic Signaling in Glioblastoma Cells and Paracrine Activation of Stromal Fibroblasts via CXCR-2

**DOI:** 10.3390/biom14040461

**Published:** 2024-04-10

**Authors:** Dimitra Manou, Maria-Angeliki Golfinopoulou, Sara Naif D. Alharbi, Hind A. Alghamdi, Fatimah Mohammed Alzahrani, Achilleas D. Theocharis

**Affiliations:** 1 Biochemistry, Biochemical Analysis and Matrix Pathobiology Research Group, Laboratory of Biochemistry, Department of Chemistry, University of Patras, 26504 Patras, Greece; dimitramanou@outlook.com (D.M.); mariantzelagolf@gmail.com (M.-A.G.); 2 Department of Chemistry, College of Science, Princess Nourah bint Abdulrahman University, P.O. Box 84428, Riyadh 11671, Saudi Arabia; snalharbi@pnu.edu.sa (S.N.D.A.); haalghamdi@pnu.edu.sa (H.A.A.); fmalzahrani@pnu.edu.sa (F.M.A.)

**Keywords:** serglycin, proteoglycans, extracellular matrix, tumor microenvironment, glioblastoma

## Abstract

Serglycin (SRGN) is a pro-tumorigenic proteoglycan expressed and secreted by various aggressive tumors including glioblastoma (GBM). In our study, we investigated the interplay and biological outcomes of SRGN with TGFβRI, CXCR-2 and inflammatory mediators in GBM cells and fibroblasts. SRGN overexpression is associated with poor survival in GBM patients. High SRGN levels also exhibit a positive correlation with increased levels of various inflammatory mediators including members of TGFβ signaling pathway, cytokines and receptors including CXCR-2 and proteolytic enzymes in GBM patients. SRGN-suppressed GBM cells show decreased expressions of TGFβRI associated with lower responsiveness to the manipulation of TGFβ/TGFβRI pathway and the regulation of pro-tumorigenic properties. Active TGFβRI signaling in control GBM cells promotes their proliferation, invasion, proteolytic and inflammatory potential. Fibroblasts cultured with culture media derived by control SRGN-expressing GBM cells exhibit increased proliferation, migration and overexpression of cytokines and proteolytic enzymes including CXCL-1, IL-8, IL-6, IL-1β, CCL-20, CCL-2, and MMP-9. Culture media derived by SRGN-suppressed GBM cells fail to induce the above properties to fibroblasts. Importantly, the activation of fibroblasts by GBM cells not only relies on the expression of SRGN in GBM cells but also on active CXCR-2 signaling both in GBM cells and fibroblasts.

## 1. Introduction

Isocitrate dehydrogenase wildtype (IDH-wt) glioblastoma (GBM) is the most lethal form of brain tumor, categorized as tumor Grade 4 by WHO [1]. Despite the current standard of care, which includes maximal surgical resection accompanied by chemotherapy and radiotherapy [2], the overall median survival of GBM patients remains around 15 months. GBM is characterized by intra-tumoral heterogeneity [3] with necrotic areas and elevated vascularity, as well as stem cell niches [4]. The extracellular matrix (ECM) plays multifaceted roles in physiological contexts and in diseases and can constantly be adapted to create a provisional matrix for tumor initiation and progression in the tumor microenvironment [5,6,7,8]. Proteoglycans (PGs), which are crucial components of the ECM, are implicated in tumorigenesis [9,10] and could serve as tumor therapeutic targets with potential application in GBM [11].

Serglycin (SRGN) is a pro-tumorigenic PG expressed and secreted by a variety of aggressive tumor cells, including GBM [12,13], breast [14,15,16], multiple myeloma [17,18] and nasopharyngeal cancer cells [19]. SRGN secreted in the ECM can create a chemotactic gradient by manipulating the bioavailability of its functional partners including growth factors and cytokines such as vascular endothelial growth factor (VEGF), hepatocyte growth factor (HGF), transforming growth factor β2 (TGFβ2) and chemokine (C-C motif) ligand 2 (CCL-2) [20,21]. SRGN can mediate the activation of multiple tumor-promoting cascades including immune system response [22,23], the expression and activity of proteolytic enzymes [24,25] and pro-tumorigenic signaling enhancing tumor cell properties such as invasion, metastasis, stemness, epithelial to mesenchymal transition (EMT) and ECM remodeling [21]. It can also bilaterally manipulate the behavior of stromal cells [21]. 

SRGN is a crucial modulator of the in vitro and in vivo tumorigenic potential of highly aggressive LN-18 GBM cells, which constitutively express and secrete high levels of SRGN. After the knockdown of SRGN, LN-18^shSRGN^ cells exhibit a tight clustered morphology, reduced stemness capacity and enhanced astrocytic differentiation features. Moreover, LN-18^shSRGN^ cells are characterized by reduced tumorigenic signaling activation and attenuated expression of proteolytic and inflammatory mediators [12]. SRGN can cooperate with cytokines and growth factors to activate signaling cascades [20,21,23,26,27]. Such an example is the cooperation of SRGN with TGFβ2 to induce EMT and breast cancer cell growth, invasion and metastasis. SRGN increases TGFβ2 levels and evokes EMT by activating CD44/CREB1 signaling axis. In turn, TGFβ2 regulates SRGN expression via activation of TGFβ receptor I (TGFβRI)/p-smad2-3 axis [28]. TGFβ pathway exerts a pro-tumorigenic role in GBM, controlling cell proliferation, stemness, angiogenesis, invasion, and immunosuppression [29,30,31,32]. TGFβ targeting has also been introduced to preclinical and clinical studies for glioma including antisense TGFβ oligonucleotides, inhibitors for modulation of TGFβRs and neutralizing antibodies [31]. 

Moreover, interleukin 8 (IL-8, CXCL-8)/C-X-C motif chemokine receptor 2 (CXCR-2) signaling axis is crucial for the aggressiveness of LN-18 cells. SRGN exerts a regulatory role in the activation of IL-8/CXCR-2 and downstream signaling pathways in GBM [12] and breast cancer cells [15]. SRGN is an activator of CXCR-2 pathway by regulating the bioavailability of IL-8, one of CXCR-2 ligands, with concomitant induction of tumor features in breast cancer cells [15]. Similarly, SRGN-suppressed LN-18^shSRGN^ cells express significantly reduced amounts of IL-8 and CXCR-2, displaying reduced activation of CXCR-2 signaling compared to control SRGN-expressing LN-18^shSCR^ cells. Blocking of CXCR-2 signaling effectively controls cell migration only in control LN-18^shSCR^ cells [12]. The protein levels of CXCR-2 are in line with the degree of malignancy of gliomas and recurrence and could possibly be used as a therapeutic target [33]. CXCR-2 pathway seems also to be an important regulator of the behavior of stromal cells in the glioma tumor microenvironment, including microglia and endothelial cells [34,35].

Recent data support the presence of a mesenchymal lineage of fibroblasts in GBM and especially near perivascular regions, creating a niche together with tumor-initiating glioma stem cells (GSCs). Cancer-associated fibroblasts (CAFs) can trigger processes such as angiogenesis, ECM remodeling, EMT and invasion as well as activation of tumor-promoting cascades to GSCs and M2 polarization of macrophages [36]. A high CAF population is in line with poor prognosis and stemness of gliomas. CAFs seem to control important pathways for tumor progression including EMT, hypoxia, inflammatory response, IFN-γ response and NF-κB-mediated TNFα signal transduction. Immunological characteristics of glioma are also associated with CAFs. The high-risk subtype is associated with a high proportion of CAFs, high stemness score and is enriched in immunosuppressive cells including Tregs and M2 macrophages [37]. Biomarkers of systemic inflammation are also associated with prognosis and overall survival in GBM [38]. The highly immunosuppressive tumor microenvironment in GBM is related to recurrent and poor prognosis [39,40] and can be driven by chronic inflammation [41]. CAFs via secretion of immune-related cytokines in high-risk subtypes are correlated with tumor-associated macrophages. It is possible that the secretion pattern of CAFs can be used as diagnostic and prognostic index of gliomas [37]. To emphasize, the overall ECM signature can collectively be utilized as a cancer therapeutic tool [42].

In our study, we investigated the interplay and biological outcomes of SRGN with cytokines in GBM cells and fibroblasts. SRGN regulates TGFβRI expression and response in GBM cells. SRGN-suppressed GBM cells are less responsive to the manipulation of TGFβRI pathway and the induction of pro-tumorigenic properties. SRGN expression is also essential to GBM cells to regulate the paracrine activation of fibroblasts. This is mediated most likely by the production of soluble factors that requires the activation of CXCR-2 signaling both in GBM cells and fibroblasts.

## 2. Materials and Methods 

### 2.1. Cell Cultures and Reagents

LN-18 GBM cell line was obtained from the American Type Culture Collection (ATCC). The transduction with shRNA lentiviral particles against SRGN was conducted and generated LN-18^shSRGN^ cells with 99% suppressed levels of SRGN accompanied by lack of its secretion in the medium, compared to LN-18^shSCR^ cells that expressed and secreted SRGN in high levels, as previously described [12]. LN-18^shSRGN^ and LN-18^shSCR^ cells were cultured in DMEM supplemented with 5% (*v*/*v*) Fetal Bovine Serum (FBS), 1 mM sodium pyruvate, 2 mM L-glutamine and a cocktail of antimicrobial agents (100 IU/mL penicillin, 100 μg/mL streptomycin, 10 μg/mL gentamycin sulfate and 2.5 μg/mL amphotericin B). The AG1523 human dermal primary fibroblasts were obtained from a 3-day-old infant and kindly provided by Dr. D. Kletsas, Institute of Biosciences and Applications, N.C.S.R. “Demokritos”. Fibroblasts were cultured in DMEM High Glucose, supplemented with 15% (*v*/*v*) FBS and 100 IU/mL penicillin and 100 μg/mL streptomycin in a humidified 95% air/5% CO_2_ incubator at 37 °C. Reagents for cell culture were supplied by Biosera, France. The specific inhibitor SB225002 of CXCR-2 (iCXCR-2) was supplied from Sigma-Aldrich, while the inhibitor GW6604 of TGFβRI (iTGFβRI) was supplied by American Custom Chemicals Corp. The human recombinant growth factor TGFβ1 was supplied by Peprotech and diluted in 0.1% BSA-4 mM HCl.

### 2.2. Generation of Culture Media 

LN-18^shSCR^ and LN-18^shSRGN^ cells were seeded in 100 mm dishes and incubated with complete medium for 24 h followed by a 4 h starvation period in serum-free (SF) medium. Afterwards, cells were incubated with SF medium for 72 h either in the absence or the presence of 3 µM iTGFβRI or 1 µM iCXCR-2 dissolved in DMSO or equal volume of DMSO. Inhibitors and DMSO were added at 0 and 36 h. Simultaneously, SF medium without cells was incubated for 72 h as a control medium for the incubation of fibroblasts. After the final incubation of 72 h, culture media (CM) were collected and centrifuged at 3000 rpm for 3 min. 

### 2.3. RNA Isolation, cDNA Synthesis and Real-Time qPCR Analysis

To investigate the role of the TGFβ pathway, LN-18^shSCR^ and LN-18^shSRGN^ cells were seeded in 60 mm dishes and incubated with complete medium for 24 h followed by overnight starvation in SF medium. Then, cells were incubated with SF medium for 72 h either in the presence of 3 µM iTGFβRI dissolved in DMSO or TGFβ1 at final concentration 5 ng/mL or equal volume of DMSO, which were added at 0 and 36 h at the respective dishes.

For the investigation of the effect of the CM of LN-18^shSCR^ and LN-18^shSRGN^ cells in fibroblasts, fibroblasts were seeded in 6-well plates and incubated with complete medium for 16 h followed by an incubation period of 4 h with medium supplemented with 2% FBS. Then, fibroblasts were incubated for 48 h with either the CM of LN-18^shSCR^ or LN-18^shSRGN^ cells supplemented with 2% FBS or control medium (DMEM) supplemented with 2% FBS.

Cells were then proceeded for total RNA extraction using NucleoSpin^®^ RNA kit (Macherey-Nagel) according to the manufacturer’s instructions. Quantification of isolated RNA was determined by absorbance measurements at 260 nm. cDNA synthesis was performed using PrimeScript^TM^ RT Reagent kit (Perfect Real-Time PCR) (TAKARA) following the manufacturer’s instructions. Real-Time qPCR analysis was conducted using the reaction mixture KAPA SYBR^®^ Fast qPCR kit Master Mix (2×) Universal (KAPABIOSYSTEMS, Wilmington, MA, USA) according to manufacturer’s instructions, gene-specific primers (Table S1) and the Rotor Gene Q (Qiagen, Germantown, MD, USA). Relative quantification of the data was obtained using the ΔΔCt method using the normalization gene GAPDH, and the fold changes were determined as 2^−ΔΔCt^. 

### 2.4. Immunoblotting

LN-18^shSCR^ and LN-18^shSRGN^ cells were seeded in 100 mm dishes and incubated with complete medium for 24 h followed by a 4 h starvation period in SF medium. Then, cells were incubated with SF medium for 48 h. Cells were lysed with lysis buffer containing 25 mM Hepes, pH 7.5, 150 mM NaCl, 5 mM EDTA, 10% (*v*/*v*) glycerol, 1% (*v*/*v*) Triton X-100 supplemented with 1x protease inhibitor cocktail (Chemicon, Millipore, CA, USA, 20-201) and 0.5 mM sodium orthovanadate (Sigma-Aldrich, St. Louis, MO, USA, S6508). Equal amounts of proteins were reduced with β-mercaptoethanol in Laemmli buffer, separated by SDS-PAGE and transferred to polyvinylidene difluoride (PVDF) membranes (Macherey-Nagel). The membranes were blocked with 5% (*w*/*v*) Bovine Serum Albumin (BSA) (Sigma-Aldrich) in TBS pH 7.4 containing 0.05% Tween-20 and then probed with primary antibodies. Detection of the bound antibodies was carried out with peroxidase-conjugated secondary goat anti-rabbit IgG (Sigma-Aldrich, A0545) or goat anti-mouse IgG (Sigma-Aldrich, A4416) and visualized by chemiluminescence (Luminata^TM^ Crescendo Western HRP Substrate, Millipore, Burlington, MA, USA). Primary antibodies used in immunoblotting analyses included TGFβRI (Abcam, ab31013, rabbit, 1:500) and β-actin antibody (Santa Cruz, AC-15, sc-69879). The density of immunoreactive bands was analyzed using Image J Software, version 1.54, where background was subtracted followed by normalization to the loading control obtained from the same gel (β-actin).

### 2.5. Cell Cycle Analysis

Fibroblasts were seeded in 6-well plates and incubated with complete medium for 16 h followed by an incubation period of 4 h with medium supplemented with 2% FBS. Afterwards, fibroblasts were incubated for 24 h with either CM of LN-18^shSCR^ or LN-18^shSRGN^ cells supplemented with 2% FBS or control medium supplemented with 2% FBS. After trypsinization and washing steps, 100 μL of cell suspension was diluted with 900 μL of DAPI (CyStain, Partec, Görlitz, Germany). Samples were incubated for 5 min at room temperature and analyzed by flow cytometry on a CyFlow Space (Partec) using a 375 nm UV laser for excitation. Cell cycle distribution was calculated using FlowMax software version 2.3 (Partec). 

### 2.6. Cell Proliferation Assay

To investigate the role of the TGFβ pathway to affect cell proliferation, LN-18^shSCR^ and LN-18^shSRGN^ cells were seeded in 24-well plates and incubated with complete medium for 24 h followed by overnight starvation in SF medium. Then, cells were incubated with SF medium for 24 h in the presence of 3 µM iTGFβRI dissolved in DMSO or equal volume of DMSO.

To investigate the role of CM from LN-18^shSCR^ and LN-18^shSRGN^ cells to induce the proliferation of fibroblasts, fibroblasts were seeded in 12-well plates and incubated with complete medium for 16 h followed by an incubation period of 4 h with medium supplemented with 2% FBS. Afterwards, fibroblasts were incubated for 24 h with either CM of LN-18^shSCR^ or LN-18^shSRGN^ cells supplemented with 2% FBS or control medium supplemented with 2% FBS.

To investigate the involvement of signaling pathways on fibroblasts’ proliferation, fibroblasts were seeded in 12-well plates and incubated with complete medium for 16 h followed by a starvation period of 4 h. Afterwards, fibroblasts were incubated for 24 h with CM of LN-18^shSCR^ or LN-18^shSRGN^ cells derived after treatment of GBM cells with either 3 µM iTGFβRI or 1 µM iCXCR-2 dissolved in DMSO or equal volume of DMSO or control DMEM medium. In another set of experiments, fibroblasts were incubated for 24 h with either CM of LN-18^shSCR^ or LN-18^shSRGN^ cells supplemented with either 3 µM iTGFβRI or 1 µM iCXCR-2 dissolved in DMSO or equal volume of DMSO or control DMEM medium.

GBM cells or fibroblasts were trypsinized and collected, centrifuged at 5000× *g* for 5 min and then counted using Trypan Blue and a hemocytometer to determine the number of living cells.

### 2.7. Wound Healing Assay

To investigate the role of the TGFβ pathway to affect the migration of GBM cells, LN-18^shSCR^ and LN-18^shSRGN^ cells were seeded in 24-well plates and incubated with complete medium for 24 h followed by overnight starvation in SF medium. Cells were then scratched using a 100 μL pipette tip. Detached cells were removed by washing, and cells were incubated for 40 min at 37 °C with SF media containing 10 μΜ of the cytostatic agent cytarabine (Sigma-Aldrich) and then were photographed [OLYMPUS CKX41 microscope with a color digital camera CMOS (SC30)]. Afterwards, cells were incubated for 24 h with SF medium in the presence of 3 µM iTGFβRI dissolved in DMSO or equal volume of DMSO. 

To investigate the role of CM from LN-18^shSCR^ or LN-18^shSRGN^ cells to induce the migration of fibroblasts, fibroblasts were seeded in 12-well plates and incubated with complete medium for 16 h followed by an incubation period of 4 h with medium supplemented with 2% FBS. Cells were then scratched using a 100 μL pipette tip. Detached cells were removed by washing and then were photographed [OLYMPUS CKX41 microscope with a color digital camera CMOS (SC30)]. Afterwards, fibroblasts were incubated for 24 h with either CM of LN-18^shSCR^ or LN-18^shSRGN^ cells or control DMEM medium, all supplemented with 2% FBS. 

To investigate the involvement of signaling pathways on fibroblasts’ migration, fibroblasts were seeded in 12-well plates and incubated with complete medium for 16 h followed by a starvation period of 4 h. Cells were then scratched using a 100 μL pipette tip. Detached cells were removed by washing and then were photographed [OLYMPUS CKX41 microscope with a color digital camera CMOS (SC30)]. Afterwards, fibroblasts were incubated for 24 h with either CM of LN-18^shSCR^ or LN-18^shSRGN^ cells derived after treatment of GBM cells with either 3 µM iTGFβRI or 1 µM iCXCR-2 dissolved in DMSO or equal volume of DMSO or control DMEM medium. In another set of experiments, fibroblasts were incubated for 24 h with either CM of LN-18^shSCR^ or LN-18^shSRGN^ cells supplemented with either 3 µM iTGFβRI or 1 µM iCXCR-2 dissolved in DMSO or equal volume of DMSO or control DMEM medium.

At the final timepoint, images were captured, and wound surface was quantified using Image J Software. The percentage of wound closure was calculated for each condition.

### 2.8. ELISA

LN-18^shSRGN^ or LN-18^shSCR^ cells were seeded in 6-well plates and incubated with complete medium for 24 h followed by overnight starvation in SF medium. Then, cells were incubated with SF medium for 72 h either in the presence of 3 µM iTGFβRI dissolved in DMSO or equal volume of DMSO, which were added at 0 and 36 h at the respective dishes. Then, the culture supernatants were collected and centrifuged at 3000 rpm for 5 min. TGFβ1 and TGFβ2 were measured using human TGFβ1 Quantikine ELISA kit (DB100B, R&D Systems Inc., Minneapolis, MN, USA) and TGFβ2 Quantikine ELISA kit (DB250, R&D Systems Inc., Minneapolis, MN, USA), respectively, according to the manufacturer’s instructions. 

LN-18^shSCR^ and LN-18^shSRGN^ cells were seeded in 60 mm dishes and incubated with complete medium for 24 h followed by overnight starvation in SF medium. Then, cells were incubated with SF medium for 48 h either in the presence of 3 µM iTGFβRI dissolved in DMSO or TGFβ1 at final concentration 5 ng/mL or equal volume of DMSO. Fibroblasts were seeded in 6-well plates and incubated with complete medium for 16 h followed by an incubation period of 4 h with medium supplemented with 2% FBS. Then, fibroblasts were incubated for 48 h with either the CM of LN-18^shSCR^ or LN-18^shSRGN^ cells supplemented with 2% FBS or DMEM supplemented with 2% FBS. Then, the culture supernatants were collected, centrifuged at 3000 rpm for 5 min and concentrated with Amicon^®^ Ultra 4 3 K centrifugal filter devices (Millipore). Interleukin 6 (IL-6) and IL-8 were measured using human IL-6 Standard TMB ELISA Development kit (Peprotech, Cranbury, NJ, USA, #900-T16) and human IL-8 ELISA (ImmunoTools, Friesoythe, Germany, #31670089) according to the manufacturer’s instructions.

### 2.9. Bioinformatic Analysis

**Figure 1:**
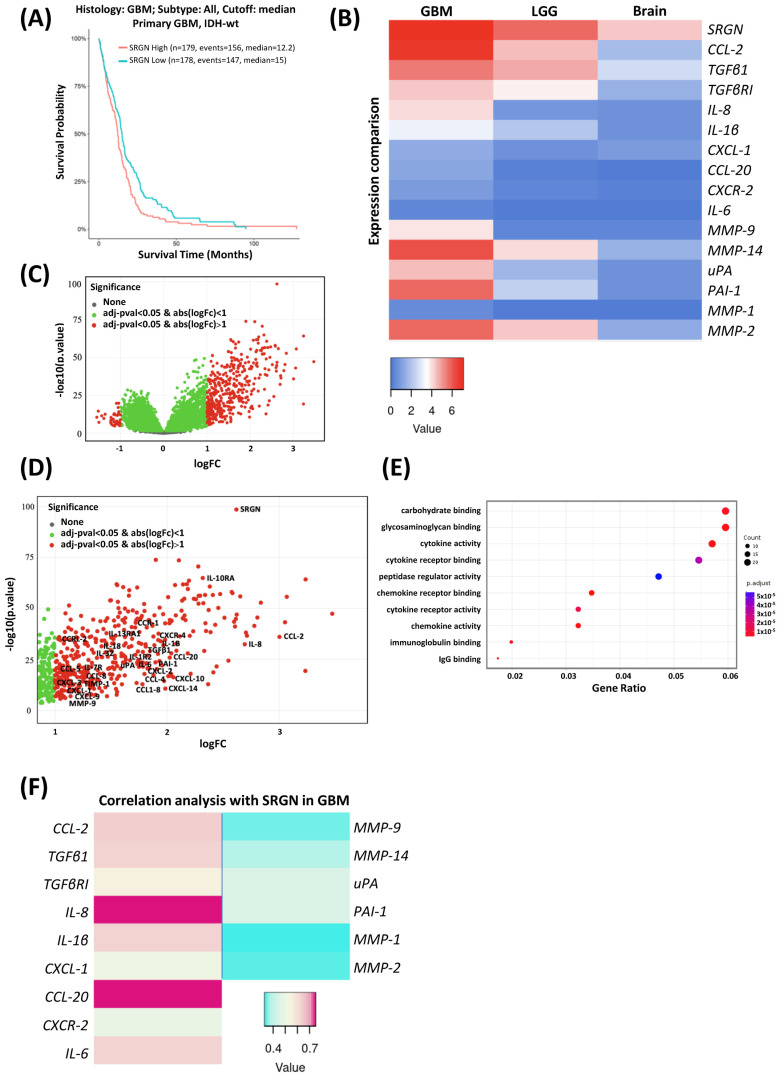
SRGN correlates with low survival and an inflammatory and proteolytic milieu in GBM. (**A**) High SRGN expression is correlated with lower survival of patients with primary IDH-wt GBM, as shown by Kaplan–Meier plot (Gliovis database) (*p* < 0.01). (**B**) Heatmap for comparison expression of SRGN, inflammatory and proteolytic enzymes, which are highly expressed in GBM tissues, compared to LGG and non-tumor brain tissues (Gepia2 database). Value indicates the log scale of transcripts per million (TPM): log_2_ (TPM+1). (**C**) Volcano plot of differentially expressed genes in GBM tissues (Gliovis database) and (**D**) magnification of the volcano plot showing the highly expressed genes, including SRGN, with emphasis to the inflammatory and proteolytic enzymes in GBM tissues. (**E**) Molecular function-related gene ontology dot plot of the highly expressed genes in GBM tissues (Gliovis database). (**F**) Correlation analysis of gene expression reveals strong positive correlation between SRGN and inflammatory soluble factors, strong-to-moderate positive correlation between SRGN and inflammatory receptors and moderate-to-weak positive correlation between SRGN and proteolytic enzymes in GBM tissues (Gepia2 database). Value indicates the correlation R value of log_2_ (SRGN TPM) plotted with log_2_ (gene of interest TPM).

For the bioinformatic analysis, public databases were used. Gliovis database was used for the Kaplan–Meier plot for the expression of SRGN in primary IDH-wt GBM tissues with parameters TCGA_GBM, platform HG-U133A and Histology GBM (Figure 1A). The same parameters were used for the generation of volcano plot and gene ontology dot plot for differential expression genes (Figure 1C,D). Heatmaps in Figure 1 were generated from data by Gepia2 database. Specifically, the parameters for Figure 1B were multiple genes comparison, GBM and LGG datasets, match TCGA normal and GTEx data, and for Figure 1F, they were correlation analysis, Pearson correlation coefficient, GBM tumor selection for TGCA tumor, brain cerebellar hemisphere and brain cerebellum selection for GTEx normal comparison. The heatmap in Figure 5A was generated from data by Timer2 using the EPIC database for cancer-associated fibroblasts. 

### 2.10. Statistical Analysis

For each assay, individual experiments were conducted at least three times. Data in diagrams are expressed as mean ± standard deviation (SD). Statistically significant differences were evaluated using an unpaired two-tailed *t*-test. GraphPad Prism 10 (GraphPad Software) was used for statistical analyses and graphs. Statistically significant differences are indicated by bars and asterisk: * (*p* ≤ 0.05).

## 3. Results

### 3.1. Serglycin Expression Is Associated with Low Survival and an Inflammatory Milieu in Glioblastoma

SRGN is correlated with the aggressive phenotype and the survival of patients in a variety of tumors. IDH-wt GBM is not an exception, as high expression of SRGN is correlated with lower survival of patients with primary GBM (Figure 1A). SRGN could act as a carrier of multiple inflammatory mediators and plays a crucial role in inflammation [20]. SRGN and several inflammatory molecules and proteolytic enzymes and inhibitors including TGFβ1, TGFβRI, IL-8, CCL-2, IL-1β, chemokine (C-X-C motif) ligand 1 (CXCL-1), IL-6, CCL-20, CXCR-2, matrix metalloproteinase 14 (MMP-14), matrix metalloproteinase 9 (MMP-9), matrix metalloproteinase 2 (MMP-2), matrix metalloproteinase 1 (MMP-1), urokinase-type plasminogen activator (uPA) and plasminogen activator inhibitor 1 (PAI-1) are highly expressed in GBM compared to low-grade gliomas (LGG) and non-tumor brain tissues (Figure 1B and Table S2). The gene expression of a variety of these factors is significantly upregulated together with SRGN in GBM tissues (Figure 1C,D). The gene ontology for the molecular function annotation of the highly expressed genes in GBM tissues revealed gene clusters associated with cytokine and chemokine activity, peptidase regulator activity, cytokine and chemokine receptor binding, cytokine receptor activity and carbohydrate and glycosaminoglycan binding (Figure 1E). Moreover, SRGN expression in GBM exhibits a strong positive correlation with the expression of inflammatory ligands such as IL-8, CCL-20, CCL-2, IL-6, IL-1β, CXCL-1 and TGFβ1, strong-to-moderate positive correlation with inflammatory receptors including TGFβRI and CXCR-2 and moderate-to-weak positive correlation with proteases and inhibitors such as PAI-1, uPA, MMP-14, MMP-9, MMP-2 and MMP-1 (Figure 1F and Table S3).

### 3.2. Serglycin Suppression Perturbs TGFβRI Pro-Tumorigenic Signaling in Glioblastoma Cells

**Figure 2:**
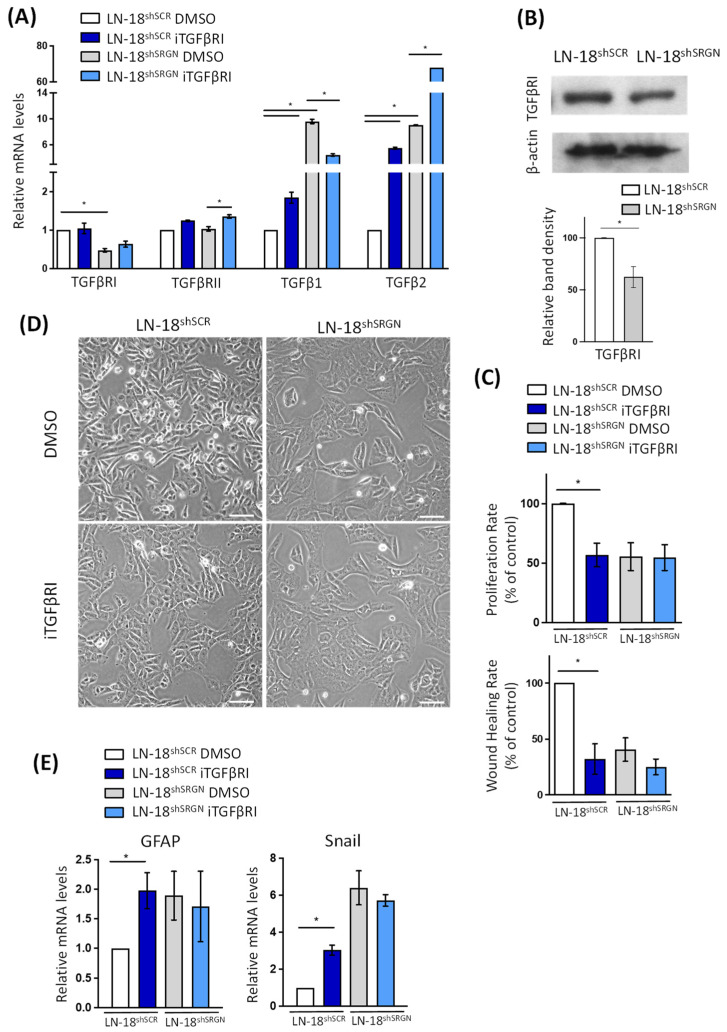
SRGN-suppressed LN-18^shSRGN^ cells are less responsive to the inhibition of TGFβRI signaling. (**A**) mRNA levels of mediators of TGFβ signaling pathway in LN-18^shSCR^ and LN-18^shSRGN^ cells in the presence or absence of iTGFβRI. (**B**) Protein levels of TGFβRI in LN-18^shSCR^ and LN-18^shSRGN^ cells. The density of immunoreactive bands was analyzed using Image J Software followed by normalization to the loading control (β-actin). (**C**) Proliferation and migration of LN-18^shSCR^ and LN-18^shSRGN^ cells in the presence or absence of iTGFβRI. (**D**) Phase-contrast microscopy images of LN-18^shSCR^ and LN-18^shSRGN^ cells treated with iTGFβRI or DMSO. Scale bar: 100 μΜ. (**E**) mRNA levels of astrocytic differentiation markers GFAP and Snail in LN-18^shSCR^ and LN-18^shSRGN^ cells in the presence or absence of iTGFβRI. Statistically significant differences are displayed by bars and asterisk * (*p* < 0.05).

Taking into consideration that TGFβ signaling exerts a potent pro-tumorigenic role in GBM cells controlling their growth, stemness and spread [29,30,31,32,43], we went to investigate the impact of SRGN suppression on the expression of TGFβRI/II and TGFβ1/2. We found that only the mRNA levels of TGFβRI were significantly decreased in LN-18^shSRGN^ compared to LN-18^shSCR^ GBM cells (Figure 2A). Immunoblot analysis confirmed the reduced protein levels of TGFβRI in LN-18^shSRGN^ (Figure 2B). In contrast, the mRNA levels of both TGFβ1 and TGFβ2 were markedly increased in LN-18^shSRGN^ (Figure 2A). The expression of TGFβ1 was upregulated in LN-18^shSCR^ and suppressed in LN-18^shSRGN^ GBM cells after treatment with iTGFβRI (Figure 2A). Elevated TGFβ1 protein levels were detected in the culture media of LN-18^shSRGN^ cells, whereas TGFβ1 secretion was diminished in LN-18^shSRGN^ cells after treatment with iTGFβRI (Figure S1). Interestingly, the expression of TGFβ2 was upregulated in LN-18^shSRGN^ and LN-18^shSCR^ GBM cells after treatment with iTGFβRI (Figure 2A). TGFβ2 secretion in the culture media of LN-18^shSRGN^ and LN-18^shSCR^ GBM cells was not detected in any case (Figure S1). Treatment of LN-18^shSCR^ and LN-18^shSRGN^ cells with iTGFβRI markedly reduced the ability of control LN-18^shSCR^ cells to proliferate and migrate, whereas these properties were not affected in LN-18^shSRGN^ cells, suggesting that a constitutive active TGFβRI signaling occurs only in control LN-18^shSCR^ cells (Figure 2C and Figure S2). The inhibition of TGFβRI in control LN-18^shSCR^ cells induced morphological alterations compatible with astrocytic differentiation (Figure 2D). Control LN-18^shSCR^ cells after treatment with iTGFβRI appeared as clusters forming tight aggregates, losing the typical aggressive phenotype of individual, spindle-shaped LN-18^shSCR^ cells resembling LN-18^shSRGN^ cells that have adopted an astrocytic less-aggressive phenotype [12]. Control LN-18^shSCR^ cells treated with iTGFβRI exhibited elevated expression of specific astrocytoma markers such as GFAP and Snail (Figure 2E). The expression of SRGN seems to be essential for the proper activation of TGFβRI signaling in LN-18 GBM cells to control cell phenotype and functions.

**Figure 3:**
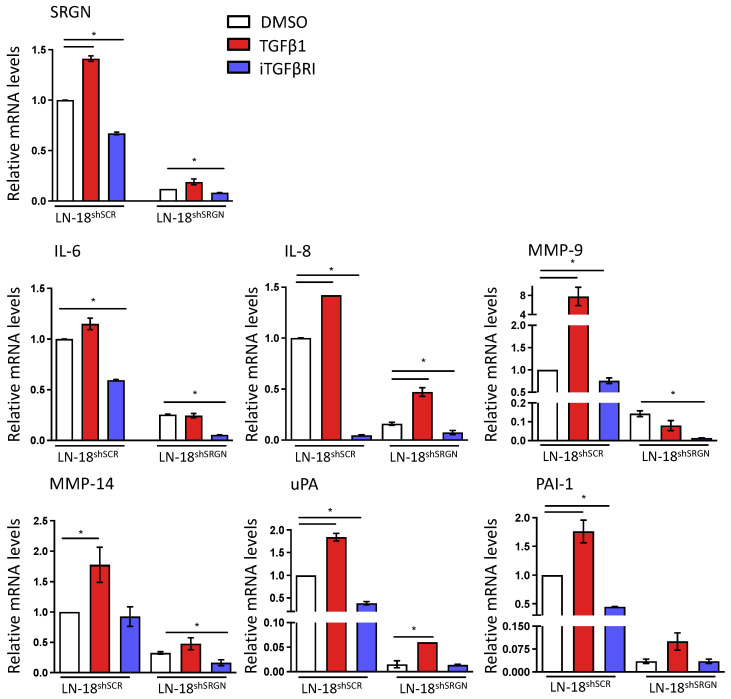
TGFβ signaling affects mainly the inflammatory and proteolytic potential of LN-18^shSCR^ cells. mRNA levels of SRGN, inflammatory and proteolytic molecules in LN-18^shSCR^ and LN-18^shSRGN^ cells after treatment with either TGFβ1 or iTGFβRI. Statistically significant differences are displayed by bars and asterisk * (*p* < 0.05).

Treatment of LN-18^shSCR^ and LN-18^shSRGN^ GBM cells with TGFβ1 to trigger TGFβRI signaling markedly induced the expression of SRGN, IL-8, MMP-9, MMP-14, uPA and PAI-1 in control LN-18^shSCR^ cells, whereas only a minor induction in the expression of IL-8 and uPA was noticed in LN-18^shSRGN^ cells (Figure 3 and Figure S3). LN-18^shSRGN^ cells show a significantly reduced expression of IL-8, IL-6, MMP-9, MMP-14, uPA and PAI-1 compared to control LN-18^shSCR^ cells. Blocking of endogenous activation of TGFβRI signaling with iTGFβRI significantly reduced the expression of several inflammatory and proteolytic mediators in both LN-18^shSCR^ and LN-18^shSRGN^ cells (Figure 3 and Figure S3). This suggests a regulatory role for TGFβRI signaling on the expression of these mediators in GBM cells that is partially regulated by the presence of SRGN.

### 3.3. Serglycin Is Involved in the Paracrine Activation of Fibroblasts by Glioblastoma Cells

**Figure 4:**
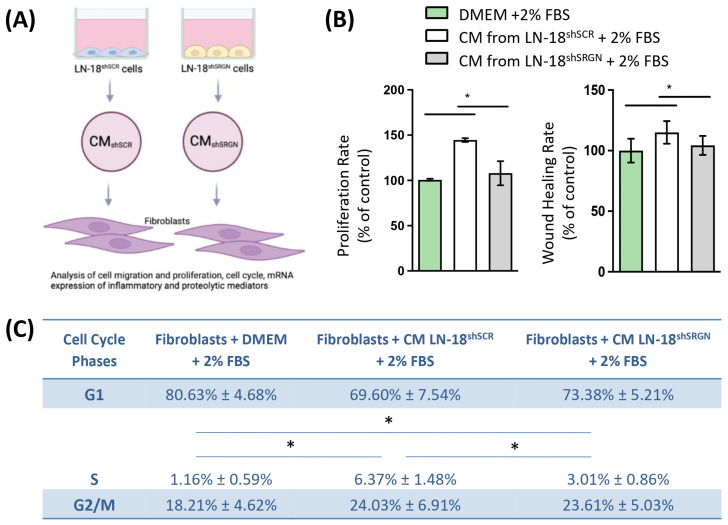
SRGN expression in LN-18 cells is required to activate fibroblasts in a paracrine manner. (**A**) Schematic illustration of the experimental approach. (**B**) Proliferation, migration and (**C**) cell cycle analysis of fibroblasts treated with either culture media (CM) from LN-18^shSCR^ or LN-18^shSRGN^ cells or control media all supplemented with 2% FBS. Statistically significant differences are displayed by bars and asterisk * (*p* < 0.05).

Considering that suppression of SRGN expression in GBM cells potently affected the expression of IL-6, IL-8 and TGFβ1 isoform, which are potent signaling molecules that mediate tumor cell–stroma interplay and can regulate the phenotype of stromal cells, we went to investigate the capacity of LN-18^shSCR^ and LN-18^shSRGN^ cells to activate stromal fibroblasts. Culture media derived by LN-18^shSCR^ and LN-18^shSRGN^ GBM cells were collected. Fibroblasts were cultured in the presence of DMEM containing 2% FBS or culture media derived from GBM cells supplemented with 2% FBS, and their proliferation and migration capacity was examined. Fibroblasts cultured in the presence of culture medium derived by control LN-18^shSCR^ cells proliferated faster and migrated more than fibroblasts cultured with either media derived by LN-18^shSRGN^ cells or DMEM supplemented with 2% FBS (Figure 4A,B and Figure S4). Additionally, the cell cycle distribution of fibroblasts was affected by the culturing conditions as expected. The percentage of cells found in the S-phase was markedly increased when fibroblasts were cultured in the presence of culture medium derived by control LN-18^shSCR^ cells, whereas culture medium derived by LN-18^shSRGN^ cells affected S-phase distribution to a lesser extent (Figure 4C). 

**Figure 5:**
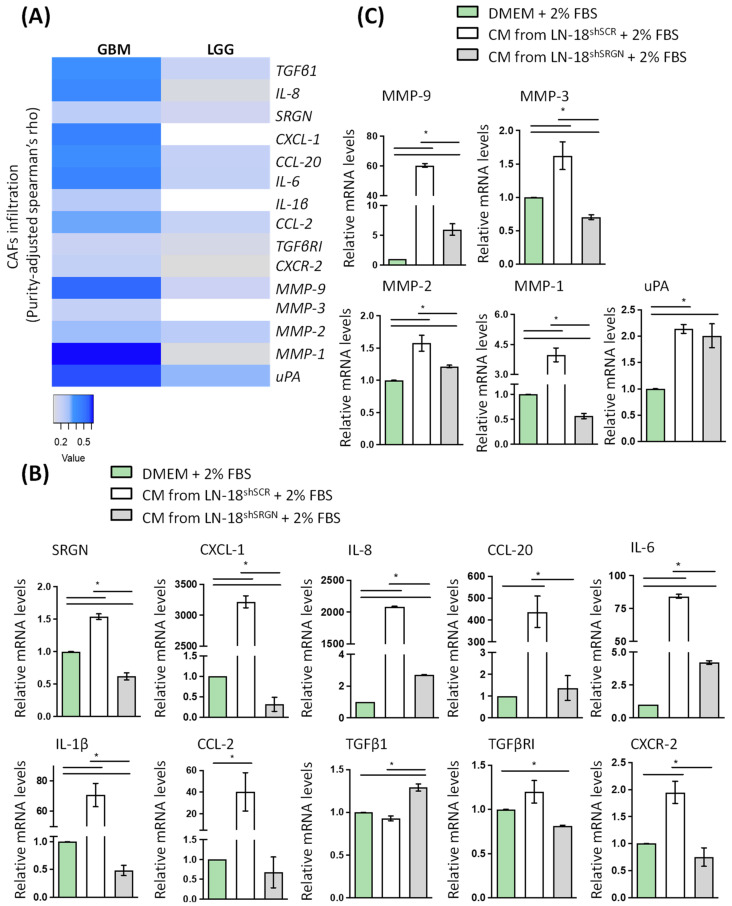
SRGN is important for LN-18 cells to induce inflammatory and proteolytic cascades in fibroblasts, which are linked with their infiltration ability. (**A**) Significant positive correlation of the expression of SRGN, inflammatory and proteolytic molecules with the infiltration of CAFs in GBM (Timer2, EPIC database). White boxes indicate a non-significant correlation. Value indicates Spearman’s ρ for positive correlation (*p* < 0.05, ρ > 0) of gene of interest with the infiltration of CAFs in GBM or LGG. (**B**) mRNA levels of inflammatory mediators and (**C**) proteolytic enzymes in fibroblasts treated with either culture media (CM) from LN-18^shSCR^ or LN-18^shSRGN^ cells or control media all supplemented with 2% FBS. Statistically significant differences are displayed by bars and asterisk * (*p* < 0.05).

Activation of stromal cells in the tumor microenvironment and generation of CAFs is a hallmark in tumor progression. CAFs secrete matrix components, proteolytic enzymes and numerous growth factors and cytokines, remodeling ECM and creating a pro-tumorigenic milieu. The infiltration of CAFs in GBM and to a lesser extent in LGG is associated with the expression of various inflammatory mediators and proteolytic enzymes (Figure 5A). We went to examine the ability of GBM cells to induce the expression of these molecules in fibroblasts. When fibroblasts cultured in the presence of culture medium derived by LN-18^shSCR^ cells, a tremendous induction in the expression of CXCL-1, IL-8, CCL-20, IL-6, IL-1β, CCL-2 and MMP-9 as well as a significant upregulation in the expression of MMP-2, MMP-3, MMP-1, uPA and CXCR-2 by fibroblasts were detected (Figure 5B,C and Figure S5). In contrast, the culture medium derived by LN-18^shSRGN^ cells failed to induce remarkably the expression of the inflammatory mediators and proteolytic enzymes, and only minor stimulatory effect was found in the expression of MMP-2, MMP-9, uPA, IL-6, IL-8 and TGFβ1 (Figure 5B,C and Figure S5). These data again suggest that SRGN is a key molecule of GBM cells that not only drives their tumorigenic potential but is also involved in their crosstalk with stromal cells. 

### 3.4. Active CXCR-2 Signaling Is Essential for Glioblastoma Cells–Fibroblasts Crosstalk and Activation

**Figure 6:**
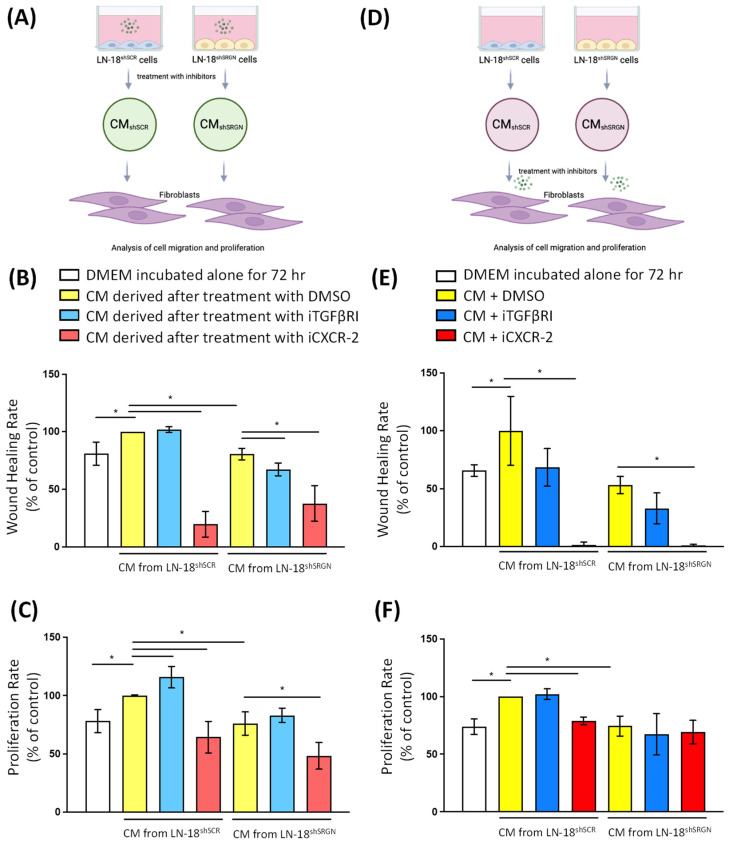
CXCR-2 pathway is critical for the crosstalk of GBM cells and fibroblasts. (**A**) Schematic illustration of the experimental approach, in which LN-18^shSCR^ and LN-18^shSRGN^ cells were treated with iTGFβRI or iCXCR-2, and their culture media (CM) were collected and tested for their ability to affect functional properties of fibroblasts, such as (**B**) migration and (**C**) proliferation. (**D**) Schematic illustration of the experimental approach, in which CM collected from LN-18^shSCR^ and LN-18^shSRGN^ cells were used to treat fibroblasts in the presence of iTGFβRI or iCXCR-2 to study the effect of respective signaling pathways on functional properties of fibroblasts, such as (**E**) migration and (**F**) proliferation. Statistically significant differences are displayed by bars and asterisk * (*p* < 0.05).

Then, we cultured LN-18^shSCR^ and LN-18^shSRGN^ cells, in the absence or in the presence of iTGFβRI and iCXCR-2, to block the autocrine constitutive activation of TGFβRI and CXCR-2 signaling pathways in GBM cells, and culture media were collected in order to study the ability of the above pathways to affect soluble factors produced by GBM cells and influence fibroblasts’ functions. Then, we cultured fibroblasts with the above culture media collected by GBM cells treated with iTGFβRI and iCXCR-2, and fibroblasts’ proliferation and migration were examined. The culture medium derived by LN-18^shSRGN^ cells after inhibition of autocrine activation of TGFβRI signaling axis slightly reduced fibroblasts’ migration (Figure 6A–C). In contrast, the culture medium derived by both LN-18^shSCR^ and LN-18^shSRGN^ cells after inhibition of autocrine activation of CXCR-2 signaling potently reduced fibroblasts’ proliferation and migration (Figure 6A–C). 

Then, we went to investigate the contribution of TGFβRI and CXCR-2 signaling pathways in fibroblasts regarding their activation by culture media derived by GBM cells. We cultured fibroblasts in the presence of culture media derived by either LN-18^shSCR^ or LN-18^shSRGN^ cells and then supplemented with iTGFβRI and iCXCR-2 to block the activation of TGFβRI and CXCR-2 signaling pathways in fibroblasts. Inhibition of TGFβRI signaling in fibroblasts treated with culture media derived by either LN-18^shSCR^ or LN-18^shSRGN^ cells did not exhibit any effect on fibroblasts’ proliferation and migration (Figure 6D–F). In contrast, inhibition of CXCR-2 signaling diminished fibroblasts’ migration induced by culture medium derived by control LN-18^shSCR^ cells below basal levels of unstimulated fibroblasts. Similarly, inhibition of CXCR-2 signaling significantly reduced fibroblasts’ proliferation induced by culture medium derived by control LN-18^shSCR^ cells. Treatment with iCXCR-2 also markedly diminished fibroblasts’ migration treated with culture medium derived by LN-18^shSRGN^ but did not have any effect on fibroblasts’ proliferation (Figure 6D–F). These data not only reveal that autocrine CXCR-2 signaling pathway activation in GBM cells is involved in the latter paracrine activation of stromal fibroblasts but also the direct activation of CXCR-2 signaling in fibroblasts is essential to regulate their functional properties. 

## 4. Discussion

This study investigates the cooperation of SRGN with TGFβRI and CXCR-2 signaling pathways in GBM. Specifically, the expression of SRGN regulates the activation of TGFβRI axis to control the aggressive behavior of GBM cells. SRGN is also crucial for GBM cells to communicate with stromal cells via soluble factors. The communication of GBM cells with fibroblasts is controlled by CXCR-2 signaling in both cell types. Overall, SRGN is a crucial factor in the tumor microenvironment controlling inflammatory and proteolytic remodeling molecules that contribute to the aggressive nature of GBM, as well as the infiltration and activation of stromal fibroblasts. 

Our study shows that GBM tissues are enriched in SRGN, inflammatory molecules and proteolytic enzymes and inhibitors including TGFβ1, TGFβRI, IL-8, CCL-2, IL-1β, CXCL-1, IL-6, CCL-20, CXCR-2, MMP-14, MMP-9, MMP-2, MMP-1, uPA and PAI-1 compared to LGG and non-tumor brain tissues. We focused on TGFβ pathway, which is a key player in GBM progression [29,30,31,32,43,44]. We found that TGFβ pathway is mainly active in LN-18^shSCR^ cells expressing SRGN and not in LN-18^shSRGN^ cells. The use of iTGFβRI induces morphological and functional changes only in LN-18^shSCR^ cells, which from a spindle-shape morphology acquire a less elongated shape, forming tight clusters with concomitant reduction in their proliferative and migratory capacity. No respective effects are observed in LN-18^shSRGN^ cells. Moreover, the inhibition of TGFβRI induces the expression of GFAP and Snail in LN-18^shSCR^ cells, two astrocytic markers that were upregulated after the suppression of SRGN in LN-18^shSRGN^ cells [12]. The TGFβ pathway is well known for its oncogenic role in GBM as it positively regulates cellular proliferation, stemness, invasion and angiogenesis [29,30,32]. It is shown that SRGN, due to its interaction with CD44, creates a positive loop with TGFβ2, regulating cellular migration, invasion, EMT and metastasis of the triple-negative breast cancer cell line MDA-MB-231 [28]. Computational data about the predicted interacting partners of SRGN include the ligands TGFβ1/β2/β3, the receptors ΤGFβRI and ΤGFβRII as well as SMAD2 and SMAD3 [21]. TGFβ1, but also bone morphogenetic protein (BMP) pathway, can regulate the expression of Snail and the opposite. Snail possesses a binding site, next to a SMAD3 binding site, at the transcription start site of TGFB1, where applying its repressive role. The regulation of Snail through the BMP signaling results in an astrocytic fate switch in GBM cells with loss of stemness and multidrug resistance-related genes and upregulation of differentiation-related genes [30]. The expression levels of Snail and GFAP are correlated in GBM cells, and GFAP can be induced by BMP7 in a Snail-dependent manner, but the exact mechanism is still unknown [45]. TGFβ1 can also positively regulate GFAP expression as seen in astrocytes under wound healing conditions [46,47] or after the treatment of GBM cells with the growth factor [30]. 

Furthermore, the activation of TGFβ pathway triggers, mainly in LN-18^shSCR^ cells, the expression of important inflammatory mediators and proteolytic enzymes including IL-8, MMP-9, MMP-14, uPA and PAI-1, while the inhibition of TGFβ pathway induces the opposite expression pattern. LN-18^shSRGN^ cells present already lower expression levels of the above molecules due to the suppression of SRGN and show reduced sensitivity to the activation or inhibition of the TGFβ pathway, most likely due to lower expression of TGFβRI in these cells. The investigated molecules are abundant in GBM tissues and exhibit a strong positive expression correlation with SRGN. TGFβ interplays with the proteolytic cascade for bilateral regulation, as TGFβ can control the expression of MMPs and uPA [48,49,50,51,52,53,54,55,56,57], and in turn, these proteases activate the growth factor [58,59,60,61,62]. Moreover, MMPs and proteases of plasminogen activation system cooperate to create an intra-activation loop. Altogether, synergistically they can promote EMT, invasion and metastasis of cancer cells [57,63]. The crosstalk of TGFβ1 and cytokines is also well stated in the literature. For example, expression and secretion of IL-6 and TGFβ1 are interconnected in biliary tract cancer cells, strengthening invasion, EMT and chemoresistance [64]. TGFβ1 enhances IL-8 promoter activity in prostate cancer cells [65], while TGFβ1 also regulates IL-8 expression in SUM149 and MDA-MB-231, facilitating cancer stem cell expansion [66]. Our data indicate that SRGN is important for the maintenance of active TGFβ pathway, and their cooperation creates a more favorable tumor-supporting milieu for GBM cells. 

We also investigated whether the suppression of SRGN in LN-18 GBM cells interferes with the communication between GBM cells and fibroblasts. In detail, culture media (CM) from LN-18^shSCR^ and not from LN-18^shSRGN^ GBM cells lead to the activation of the proliferation and migration of treated fibroblasts. Fibroblasts activated by the CM of LN-18^shSCR^ cells exhibit an enhanced inflammatory and proteolytic potential, as shown by the enormous increase in the expression of SRGN, IL-8, IL-6, CCL-2, CCL-20, IL-1β and CXCL-1 as well as by the significant induction of CXCR-2, MMP-1, MMP-2, MMP-3, MMP-9 and uPA. Conversely, fibroblasts treated with CM from LN-18^shSRGN^ cells present only a minor induction of IL-6, IL-8, TGFβ1, MMP-2, MMP-9 and uPA. The accumulation of these molecules positively correlates with the infiltration of fibroblasts in GBM tissues. This may indicate that activated fibroblasts also contribute to the accumulation of pro-inflammatory molecules in the tumor microenvironment in GBM.

Activated fibroblasts emerged to play an important role in the stemness and inflammatory status of GBM [36,37]. The ability of SRGN to manipulate the behavior of stromal cells and the inflammatory potential in the tumor microenvironment is well stated in the literature [21]. A positive correlation between SRGN expression and number of mast cells has been described in GBM. SRGN-expressing glioma cells after co-culture with mast cells further enhance their expression of SRGN, CD44, CXCL-10, CXCL-12 and TNFα as well as EMT-related genes ZEB-1 and vimentin. Moreover, that interaction also increases the expression of IL-6 and CXCL-1 in mast cells [13]. Normal breast epithelial cell line HMLE, after knockout of SRGN, exhibits lower sensitivity to TGFβ-induced EMT, revealing that SRGN is essential at the early stages of EMT [67]. Co-culture of stromal fibroblasts with breast cancer cells results in elevated expression of SRGN and secretion of ADAMTS1 in fibroblasts and induced invasiveness of tumor cells [68]. Finally, SRGN is involved in a paracrine loop between esophageal cancer cells and fibroblasts with the last ones to be activated creating a favorable microenvironment [69]. 

We established that suppression of SRGN in LN-18^shSRGN^ cells substantially down-regulates IL-8/CXCR-2 expression and signaling that is active and important in control LN-18^shSCR^ cells to regulate tumor cell functions [12]. So, we went to investigate whether the activity of tumorigenic signaling pathways in LN-18 GBM cells, affected by the presence of SRGN, such as TGFβRI and CXCR-2, are involved in the activation of fibroblasts. The findings that CM derived by both LN-18^shSCR^ and LN-18^shSRGN^ cells, in which constitutive CXCR-2 signaling was inhibited, markedly reduced fibroblasts’ proliferation and migration suggest a potent role for this pathway in the paracrine activation of fibroblasts. In contrast, inhibition of TGFβRI in GBM cells has no effect on fibroblast activation.

Then, we examined the role of both TGFβRI and CXCR-2 signaling pathways in fibroblasts regarding their paracrine activation by GBM cells. Again, we found that only the inhibition of CXCR-2 signaling in fibroblasts strongly reduces their migration, not only alleviating the inducing effect of CM derived by LN-18^shSCR^ on these cells but also diminishing their migration much lower than basal levels of unstimulated fibroblasts. Inhibition of CXCR-2 in fibroblasts also eliminates the induction of CM derived by LN-18^shSCR^ on fibroblasts’ proliferation. In contrast, as shown by the inhibition of TGFβRI in fibroblasts, this pathway does not significantly interfere with the paracrine activation of fibroblasts by GBM cells.

CXCR-2 ligands include CXCL-1, CXCL-2, CXCL-3, CXCL-5, IL-6, CXCL-7 and IL-8 [70,71], and overall CXCLs/CXCR-2 pathway is implicated in cancer and inflammation. In the tumor microenvironment, CXCLs/CXCR-2 cascade can signal through RAS/ERK, PI3K/AKT/mTOR, PKC/p38/NF-κB, JAK/STAT3 and PLD to regulate DNA damage, senescence, angiogenesis, survival, proliferation, migration and further administration of chemokines [72]. CXCR-2 antagonists are of increasing importance as therapeutic agents for cancer and chronic inflammatory diseases [73,74], while ongoing clinical trials for CXCR-2 involve compounds such as ladarixin for diabetes 1 (NCT04628481), SX-682 for myelodysplastic syndromes (NCT04245397), metastatic pancreatic ductal adenocarcinoma (NCT04477343) and melanoma (NCT03161431) as well as autologous CXCR-2-modified CD70 CAR (8R-70CAR) T cells in GBM (NCT05353530). 

## 5. Conclusions

To conclude, our study demonstrates that the expression of SRGN is essential for TGFβ pathway to exert its oncogenic role in GBM cells most likely by regulating TGFβRI levels. Moreover, SRGN is also crucial for generating a communication platform for GBM cells to produce soluble factors to interplay with stromal fibroblasts. This crosstalk is mediated by active CXCR-2 signaling in both GBM cells and fibroblasts. In total, the regulatory role of SRGN in the tumor microenvironment is highlighted due to its involvement as supporter and amplifier of inflammatory and proteolytic response, reshaping concomitantly the cellular neighborhoods. 

## Data Availability

The data presented in this study are available in this article.

## Supplementary Materials

The following supporting information can be downloaded at: https://www.mdpi.com/article/10.3390/biom14040461/s1, File S1: Supplementary Tables and Figures; Table S1: Primer sequence used for Real-Time qPCR analysis; Table S2: Expression comparison values of genes of interest in GBM, LGG and non-tumor brain used for Fig1B heatmap; Table S3: Correlation analysis values of genes of interest with SRGN in GBM used for Fig1F heatmap; Figure S1: Serglycin knockdown increases the expression and secretion of TGFβ1; Figure S2: Representative photos for the wound healing assay shown in Figure 2C; Figure S3: TGFβ signaling affects the expression of IL-6 and IL-8 in LN-18^shSCR^ cells; Figure S4: Representative photos for the wound healing assay shown in Figure 4B; Figure S5: SRGN is important for LN-18 cells to induce inflammatory cascades in fibroblasts; File S2: Original Western Blots.
