# Supplementary material for: The Expression of Serglycin Is Required for Active Transforming Growth Factor β Receptor I Tumorigenic Signaling in Glioblastoma Cells and Paracrine Activation of Stromal Fibroblasts via CXCR-2
Source: Biomolecules. 2024 Apr 10;14(4):461. doi: 10.3390/biom14040461 (PMC11048235; doi:10.3390/biom14040461)
Supplement: Supplementary file 1 [file biomolecules-14-00461-s001.zip › File S1.pdf]

The Expression of Serglycin Is Required for Active Transforming Growth Factor β Receptor I Tumorigenic Signaling in Glioblastoma Cells and Paracrine Activation of Stromal Fibroblasts via CXCR-2

Dimitra Manou, Maria-Angeliki Golfinopoulou, Sara Naif D. Alharbi, Hind A. Alghamdi, Fatimah Mohammed Alzahrani and Achilleas D. Theocharis

Supplementary materials

Table S1 Page 2,3

Table S2 Page 4

Table S3 Page 5

Figure S1 Page 6

Figure S2 Page 7

Figure S3 Page 8

Figure S4 Page 9

Figure S5 Page 10

Table S1. **Primer sequence used for Real-Time qPCR analysis.**

Gene	Primer Sequence (5'-3')		$T_{annealing}$ (°C)
CCL-2	F	AAGATCTCAGTGCAGAGGCTCG	60
	R	TTGCTTGTCCAGGTGGTCCAT	
CCL-20	F	GTGCTGCTACTCCACCTCTG	60
	R	CGTGTGAAGCCCACAATAAA	
CXCR-2	F	CTTCACAGTCACATTCCAAGCCTC	58
	R	GCACCAGGGCAAGCTTTCTAAAC	
CXCL-1	F	CTGAGGAGCCTGCAACATGC	60
	R	TGATCTCATTGGCCATTTGCTT	
GAPDH	F	AGGCTGTTGTCATACTTCTCAT	As required
	R	GGAGTCCACTGGCGTCTT	
GFAP	F	TGCGGCTCGATCAACTCA	60
	R	GTTGGTTTCATCCTGGAGCTTCT	
IL-1β	F	GGGCAAGAAGTAGCAGTGTCTGTAAA	60
	R	AGAGAGCACACCAGTCCAAATTGA	
IL-6	F	TCCAGAACAGATTTGAGAGTAGTG	57
	R	GCATTTGTGGTTGGGTCAGG	
IL-8	F	CTCCAAACCTTTCCACCCC	58
	R	GATTCTTGGATACCACAGAGAATG	
MMP-1	F	CCTCGCTGGGAGCAAACA	60
	R	TTGGCAAATCTGGCGTGTAA	
MMP-2	F	CGTCTGTCCCAGGATGACATC	62
	R	ATGTCAGGAGAGGCCCCATA	
MMP-3	F	ATTCCATGGAGCCAGGCTTTC	60
	R	CATTTGGGTCAAACTCCAACTGTG	
MMP-9	F	TTCCAGTACCGAGAGAAAGCCTAT	60
	R	GGTCACGTAGCCCACTTGGT	
MMP-14	F	CATGGGCAGCGATGAAGTCT	60
	R	CCAGTATTTGTTCCCCTTGTAGAAGTA	
PAI-1	F	CTGACTTCACGAGTCTTTCAGACC	60
	R	CCCATGAAAAGGACTGTTCCTGTG	

SNAIL	F	CACTATGCCGCGCTCTTTC	60
	R	GCTGGAAGGTAAACTCTGGATTAGA	
SRGN	F	GTTGGCGTGCAGCTGGGAGA	60
	R	GGCTCTCCGCGTAGGATAACCTTG	
TGFβ1	F	GCCTTTCCTGCTTCTCATGG	60
	R	TCCTTGCGGAAGTCAATGTAC	
TGFβ2	F	GCGACGAAGAGTACTACGCC	60
	R	TGGCATCAAGGTACCCACAG	
TGFβRI	F	TGGCTCAGGTTTACCATTGCTT	60
	R	AACTTCTTCTCCCCGCCACT	
TGFβRII	F	AGAGACAGTTTGCCATGACCC	60
	R	ACAAGTCAGGATTGCTGGTGTT	
uPA	F	ACTACTACGGCTCTGAAGTCACCA	60
	R	GAAGTGTGAGACTCTCGTGTAGAC	

Table S2. **Expression comparison values of genes of interest in GBM, LGG and non-tumor brain used for Fig1B heatmap.**

Value indicates the log scale of transcripts per million (TPM): $\log_2(TPM+1)$.

GENE	GBM	LGG	Brain
SRGN	7.10	5.70	4.30
CCL-2	6.70	4.50	1.80
TGFβ1	5.50	4.70	2.60
TGFβRI	4.30	3.70	1.50
IL-8	4.00	0.80	0.60
IL-1β	3.20	2.10	0.60
CXCL-1	1.40	0.70	0.90
CCL-20	1.20	0.20	0.10
CXCR-2	0.90	0.30	0.20
IL-6	0.30	0.10	0.10
MMP-9	3.90	0.30	0.30
MMP-14	6.20	4.10	1.50
uPA	4.50	1.70	0.70
PAI-1	5.70	2.30	0.70
MMP-1	0.50	0.00	0.00
MMP-2	5.80	4.30	1.30

Table S3. Correlation analysis values of genes of interest with SRGN in GBM used for Fig1F heatmap.

Value indicates the correlation R value of log_2(SRGN TPM) plotted with log_2(gene of interest TPM).

GENE	Value	GENE	Value
CCL-2	0.64	***MMP-9***	0.35
TGFβ1	0.63	***MMP-14***	0.38
TGFβRI	0.55	***uPA***	0.45
IL-8	0.75	***PAI-1***	0.45
IL-1β	0.63	***MMP-1***	0.33
CXCL-1	0.51	***MMP-2***	0.34
CCL-20	0.75		
CXCR-2	0.49		
IL-6	0.63		

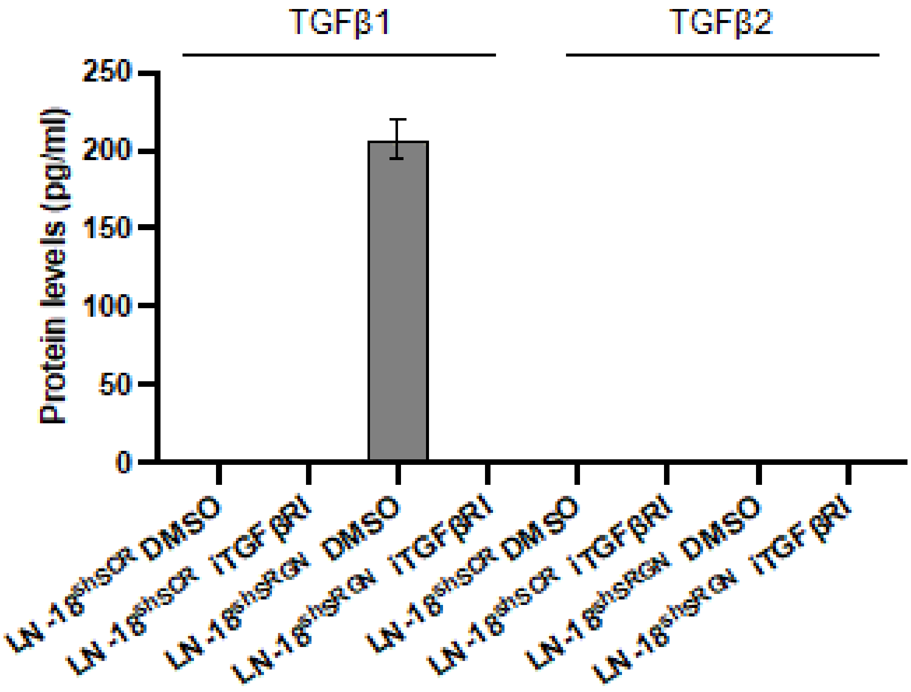

Figure S1. Serglycin knockdown increases the expression and secretion of TGFβ1.

The protein levels (pg/mL) of secreted TGFβ1 and TGFβ2 in control LN-18^{shSCR} and LN-18^{shSRGN} cells were analyzed by quantitative ELISA in culture media after incubation with SF medium for 72 h either in the presence of 3μM iTGFβRI dissolved in DMSO or equal volume of DMSO, which were added at 0 and 36 h. We have analyzed non-concentrated culture medium supernatants by using human TGFβ1 Quantikine ELISA kit (DB100B, R&D Systems Inc., Minneapolis, MN, USA) and TGFβ2 Quantikine ELISA kit (DB250, R&D Systems Inc., Minneapolis, MN, USA), respectively, according to the manufacturer's instructions. The minimum detectable dose of human TGF-β1 and TGF-β2 is 15.0 pg/mL and 7.0 pg/mL, respectively.

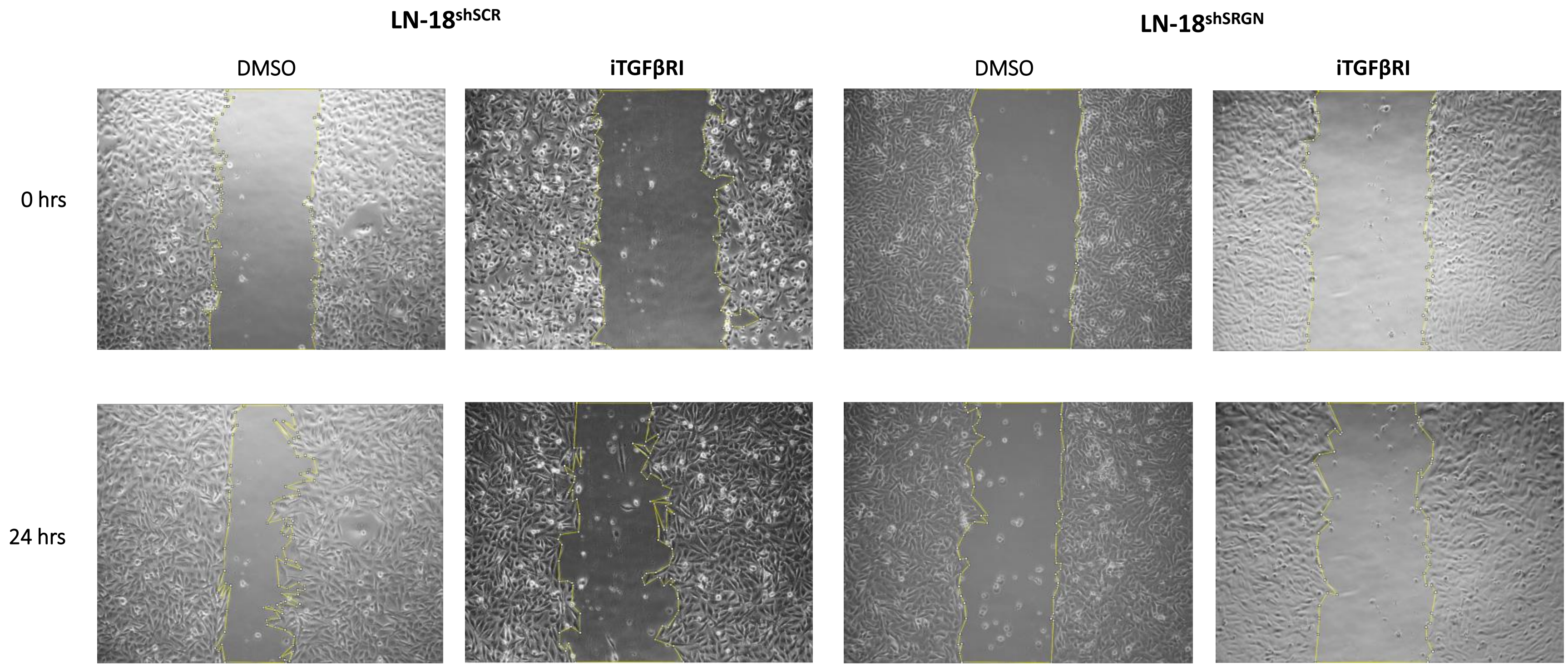

Figure S2. **Representative photos for the wound healing assay shown in Fig.2C.**

To investigate the role of the TGFβ pathway to affect the migration of GBM cells, LN-18^{shSCR} and LN-18^{shSRGN} cells were seeded in 24 well plates and incubated with complete medium for 24 h followed by overnight starvation in SF medium. Cells were then scratched using a 100 μL pipette tip. Detached cells were removed by washing and cells were incubated for 40 min at 37°C with SF media containing 10 μM of the cytostatic agent cytarabine (Sigma- Aldrich) and then were photographed (time=0 hrs). Afterwards, cells were incubated for 24 h with SF medium in the presence of 3μM iTGFβRI dissolved in DMSO or equal volume of DMSO and then were photographed (time=24 hrs).

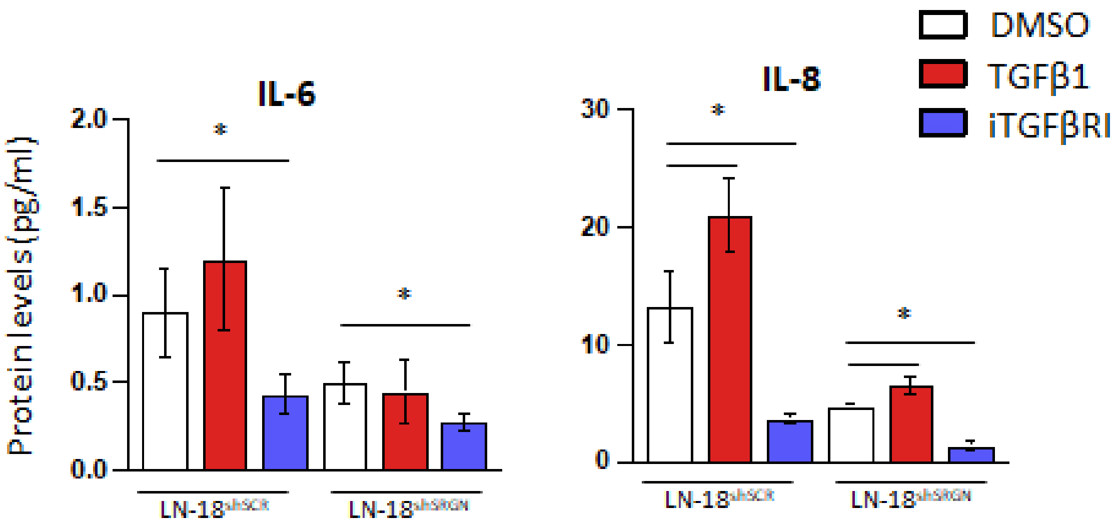

Figure S3. TGFβ signaling affects the expression of IL-6 and IL-8 in LN-18^{shSCR} cells.

Protein levels of IL-6 and IL-8 in LN-18^{shSCR} and LN-18^{shSRGN} cells after treatment with either TGFβ1 or iTGFβRI. The protein levels (pg/mL) of secreted IL-6 and IL-8 in control LN-18^{shSCR} and LN-18^{shSRGN} cells were analyzed by quantitative ELISA in culture media after incubation with SF medium for 48 h either in the presence of 3μM iTGFβRI dissolved in DMSO or TGFβ1 at final concentration 5ng/ml or equal volume of DMSO. Statistically significant differences are displayed by bars and asterisk: * ($p < 0.05$).

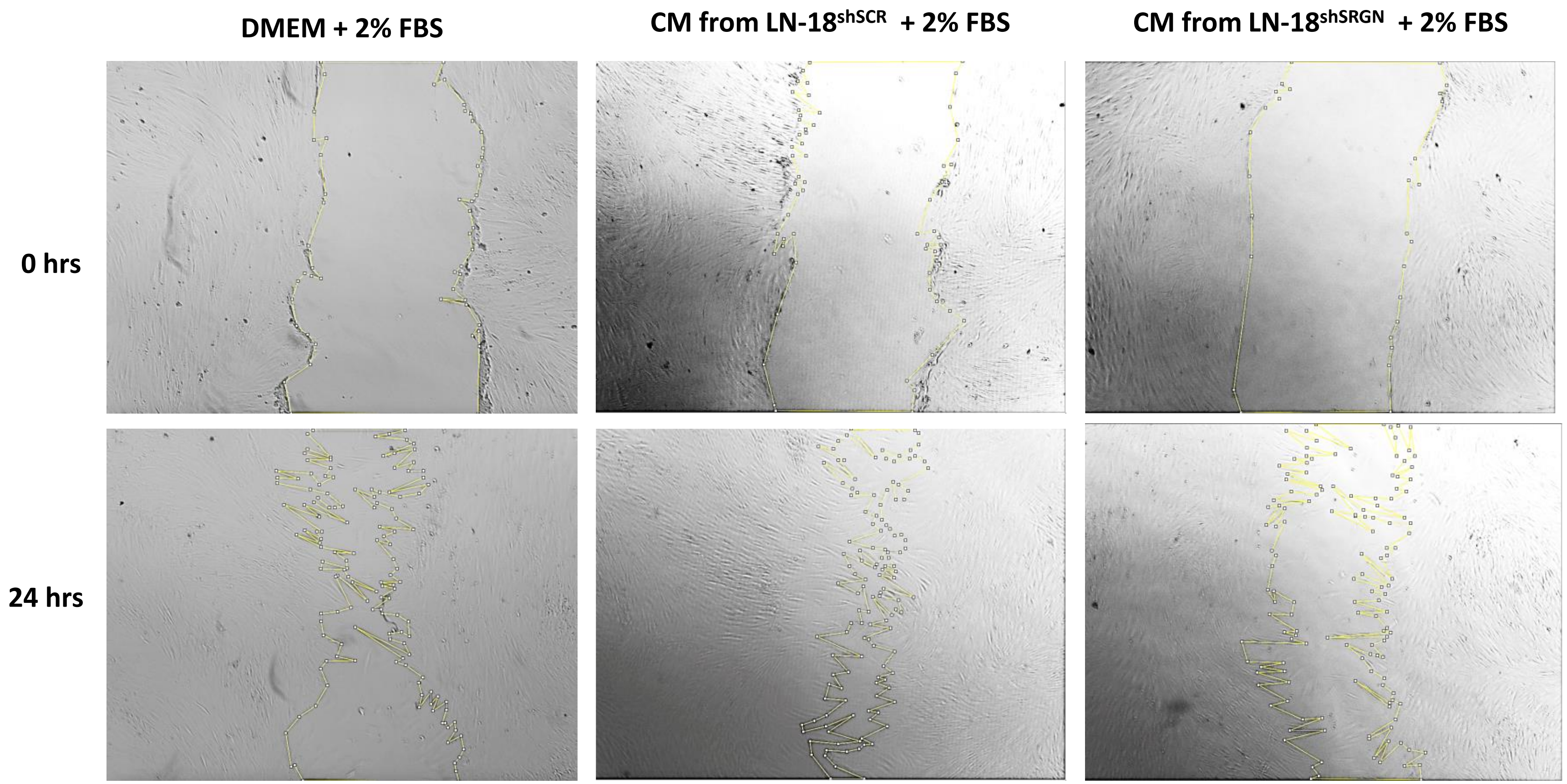

Figure S4. Representative photos for the wound healing assay shown in Fig.4B.

To investigate the role of CM from LN-18^{shSCR} or LN-18^{shSRGN} cells to induce the migration of fibroblasts, fibroblasts were seeded in 12 well plates and incubated with complete medium for 16 h followed by an incubation period of 4 h with medium supplemented with 2% FBS. Cells were then scratched using a 100 µL pipette tip. Detached cells were removed by washing and then were photographed (time=0 hrs). Afterwards, fibroblasts were incubated for 24 h either with CM of LN-18^{shSCR} or LN-18^{shSRGN} cells or control DMEM medium, all supplemented with 2% FBS and then were photographed (time=24 hrs).

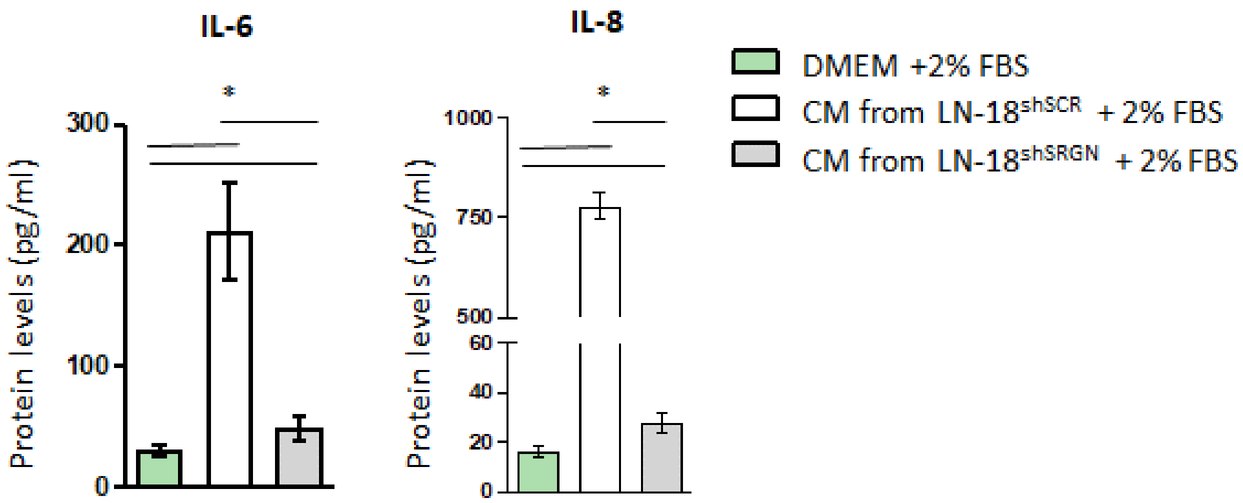

Figure S5. SRGN is important for LN-18 cells to induce inflammatory cascades in fibroblasts.

Protein levels of IL-6 and IL-8 in fibroblasts treated with either culture media (CM) from LN-18^{shSCR} or LN-18^{shSRGN} cells or control media all supplemented with 2% FBS. The protein levels (pg/mL) of secreted IL-6 and IL-8 were analyzed by quantitative ELISA in concentrated culture media. Statistically significant differences are displayed by bars and asterisk: * ($p < 0.05$).
