# Supplementary figures and images for: The Expression of Serglycin Is Required for Active Transforming Growth Factor β Receptor I Tumorigenic Signaling in Glioblastoma Cells and Paracrine Activation of Stromal Fibroblasts via CXCR-2
Source: Biomolecules. 2024 Apr 10;14(4):461. doi: 10.3390/biom14040461 (PMC11048235; doi:10.3390/biom14040461)

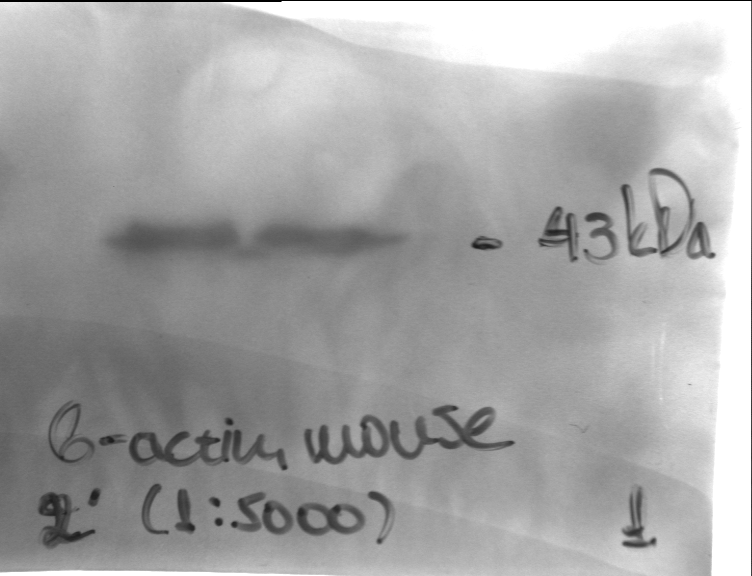

Supplement: Supplementary file 1 [file biomolecules-14-00461-s001.zip › File S2/BLOT B-ACTIN -1.TIF]

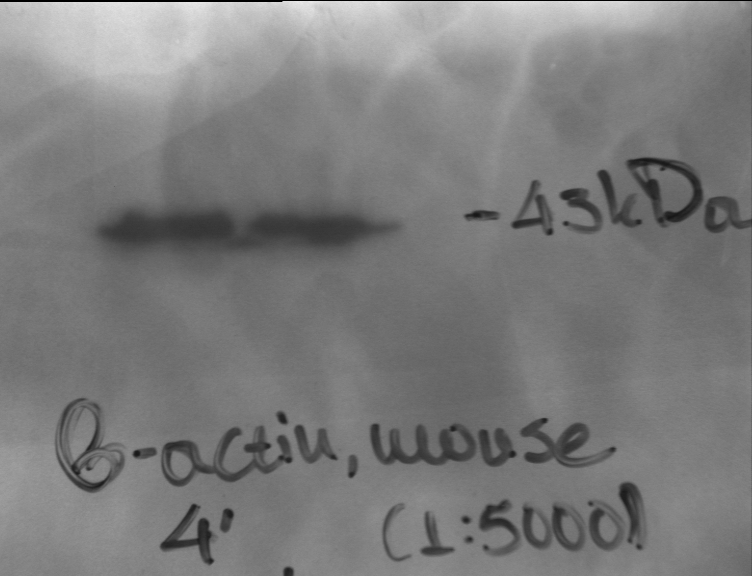

Supplement: Supplementary file 1 [file biomolecules-14-00461-s001.zip › File S2/BLOT B-ACTIN -2.TIF]

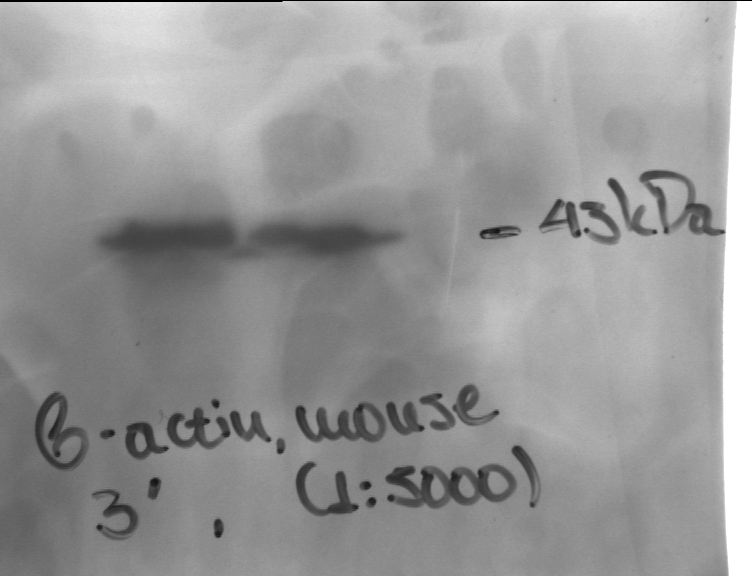

Supplement: Supplementary file 1 [file biomolecules-14-00461-s001.zip › File S2/BLOT B-ACTIN -3.TIF]

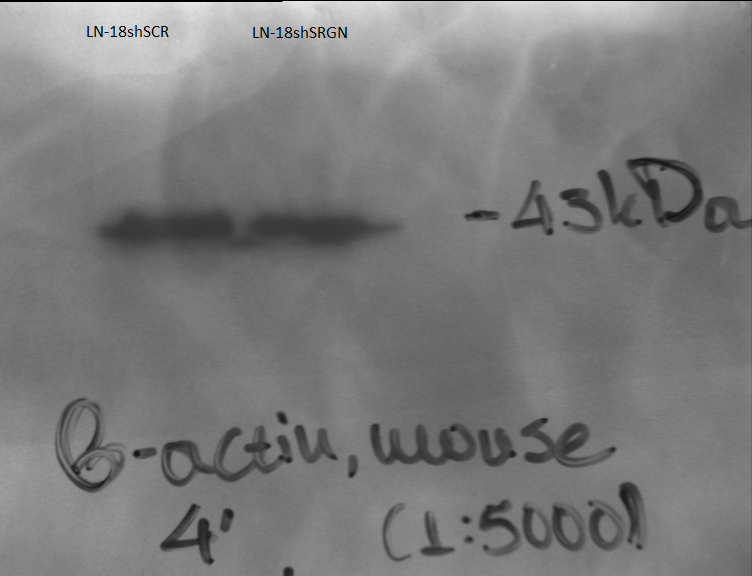

Supplement: Supplementary file 1 [file biomolecules-14-00461-s001.zip › File S2/BLOT B-ACTIN MODIFIED.tif]

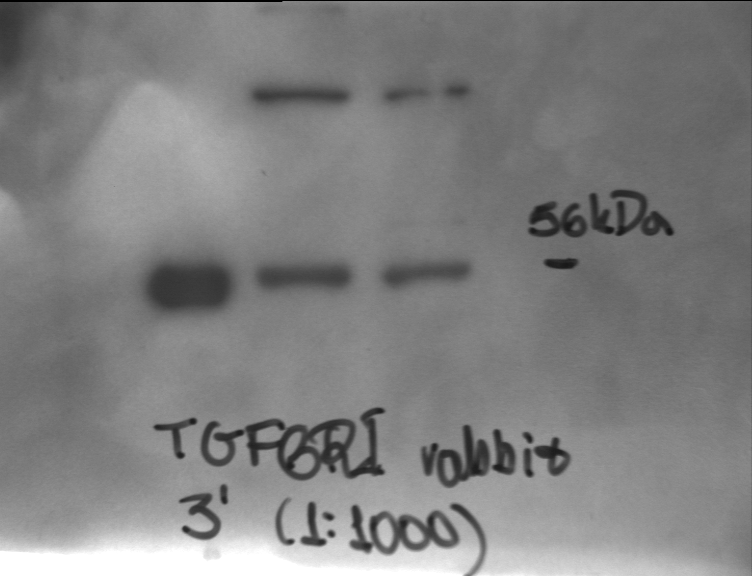

Supplement: Supplementary file 1 [file biomolecules-14-00461-s001.zip › File S2/BLOT TGFBRI -1.TIF]

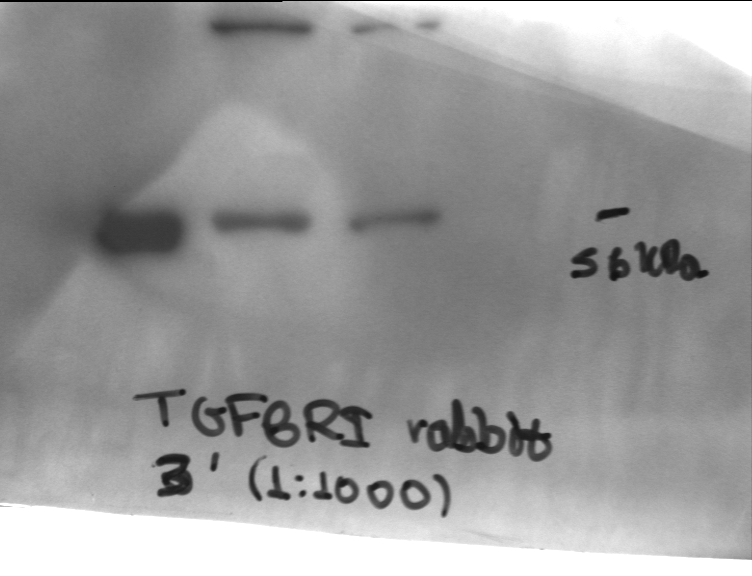

Supplement: Supplementary file 1 [file biomolecules-14-00461-s001.zip › File S2/BLOT TGFBRI -2.TIF]

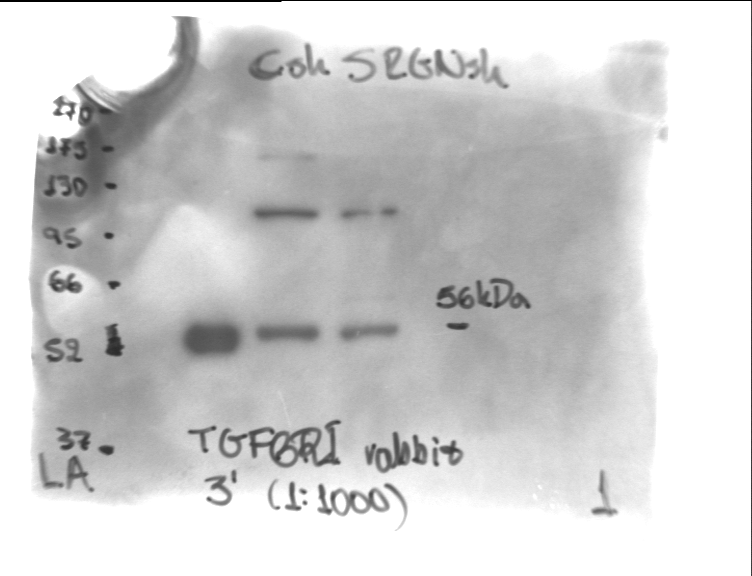

Supplement: Supplementary file 1 [file biomolecules-14-00461-s001.zip › File S2/BLOT TGFBRI -3.TIF]

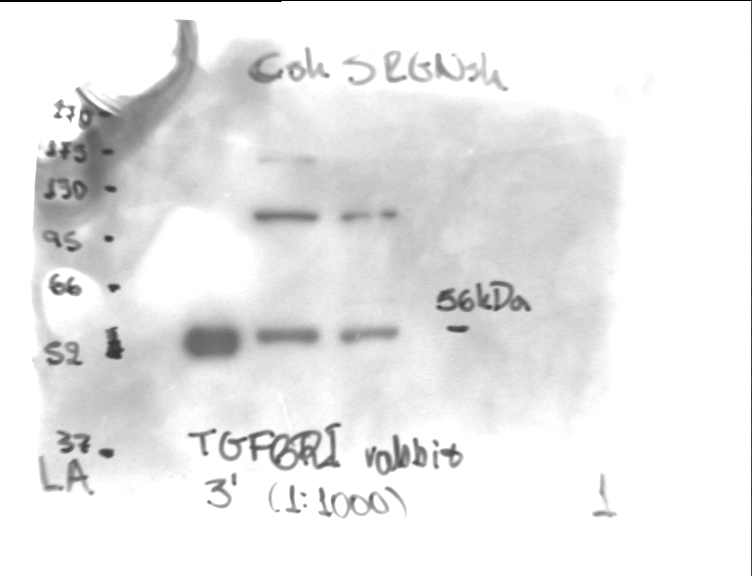

Supplement: Supplementary file 1 [file biomolecules-14-00461-s001.zip › File S2/BLOT TGFBRI -4.TIF]

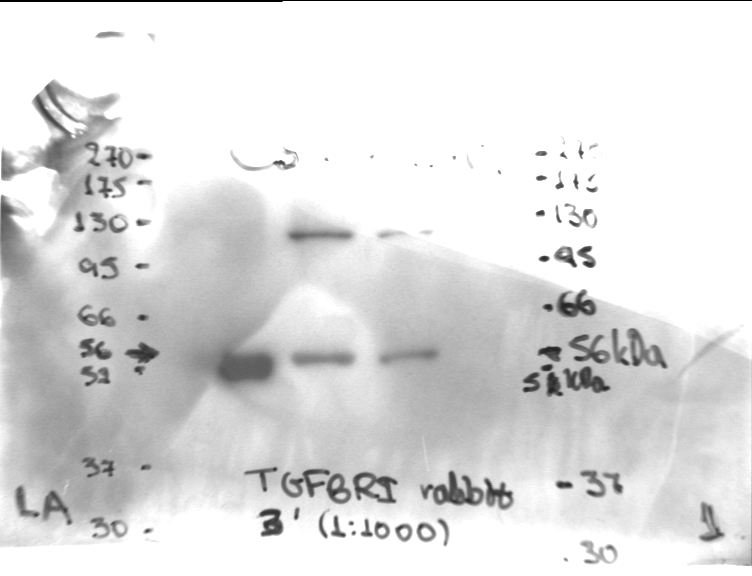

Supplement: Supplementary file 1 [file biomolecules-14-00461-s001.zip › File S2/BLOT TGFBRI -5.TIF]

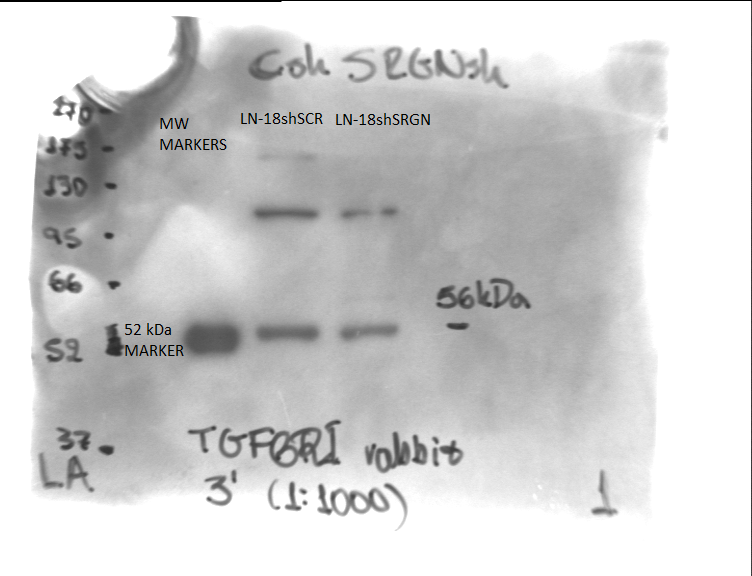

Supplement: Supplementary file 1 [file biomolecules-14-00461-s001.zip › File S2/BLOT TGFBRI MODIFIED.tif]
